# In vitro enterococcus faecalis biofilm formation on five adhesive systems

**DOI:** 10.4317/medoral.17579

**Published:** 2011-12-06

**Authors:** Pilar Baca, Márcia Furtado-Antunes de Freitas, Carmen M. Ferrer-Luque, María P. González-Rodríguez, María T. Arias-Moliz

**Affiliations:** 1 DDS, MD, PhD: Professor, Department of Preventive and Operative Dentistry, School of Dentistry. University of Granada, Granada, Spain; 2 DDS: Postgraduate Student, Department of Dental Materials, School of Dentistry. University of Sao Paulo, Sao Paulo, Brazil; 3 DDS, MD, PhD: Associate Professor, Department of Preventive and Operative Dentistry, School of Dentistry. University of Granada, Granada, Spain; 4 DDS, PhD: Associate Professor, Department of Preventive and Operative Dentistry, School of Dentistry. University of Granada, Granada, Spain; 5 DDS, PhD: Assistant Professor, Department of Microbiology, School of Dentistry. University of Granada, Granada, Spain

## Abstract

Objective: To determine the E. faecalis biofilm formation on the surface of five adhesive systems (AS) and its relationship with roughness. Study Design: The formation of E. faecalis biofilms was tested on the surface of four dual-cure AS: AdheSE DC, Clearfil DC Bond, Futurabond DC and Excite DSC and one light-cure antimicrobial AS, Clearfil Protect Bond, after 24 hours of incubation, using the MBEC high-throughput device. Results: E. faecalis biofilms grew on all the adhesives. The least growth of biofilm was on Excite DSC, Clearfil Protect Bond, and the control. Futurabond DC resulted in the greatest roughness and biofilm amount. There was a close relationship between the quantity of biofilm and roughness, except for Clearfil Protect Bond, which showed little biofilm but high roughness. Conclusion: None of the tested AS prevented E. faecalis biofilm formation, although the least quantity was found on the surface of Clearfil Protect Bond.

** Key words:**Adhesive systems, biofilm, Enterococcus faecalis, roughness.

## Introduction

Bacteria involved in persistent root canal infection would either have remained from previous treatment or would have entered by microleakage through interfacial gaps between the root canal walls and the filling material (1). A necrotic or improperly filled root canal system appears to be a habitat for enterococci, especially *Enterococcus faecalis* (2). It may be resistant to chemo-mechanical root canal treatment (3) and able to grow as biofilm on root canal walls under starving environmental conditions (4).

Sealing dentinal tubules with adhesives is an accepted treatment in endodontic and restorative dentistry, and the formation of a hybrid layer is expected to prevent coronal and apical microleakage as well as bacterial penetration in dentin tubules (5). Adhesive systems (AS) may come into direct contact with the residual bacteria in root canal dentin walls, so that the antimicrobial potential of AS is desirable. This has led to the development of AS with antibacterial components such as fluoride and 12- methacryloyloxydodecylpyridinium bromide (MDPB) (6), which has shown antibacterial activity against caries-related bacteria (7-9).

The antimicrobial activity of AS and the formation of biofilm on their surface are important factors studied mostly with *Streptococcus mutans* (10,11). To our knowledge, these aspects have not been evaluated on *E. faecalis*. The aim of this study was therefore to determine the *E. faecalis* biofilm formation on the surface of five AS —four dual-cure and one antimicrobial light-cure— and the relationship with roughness.

## Material and Methods

The AS tested comprised four dual-cure systems, AdheSE DC (Ivoclar Vivadent, Schaan, Liechtenstein), Clearfil DC Bond (Kuraray Medical Inc., Okayama, Japan), Futurabond DC (VOCO, Cuxhaven, Germany) and Excite DSC (Ivoclar Vivadent, Schaan, Liechtenstein) and one light-cure antimicrobial system, Clearfil Protect Bond (Kuraray Medical Inc., Okayama, Japan) ( Table 1). The adhesives were cured using a LED light curing unit (Bluephase, Ivoclar Vivadent, Schaan, Liechtenstein).

**Table 1:**
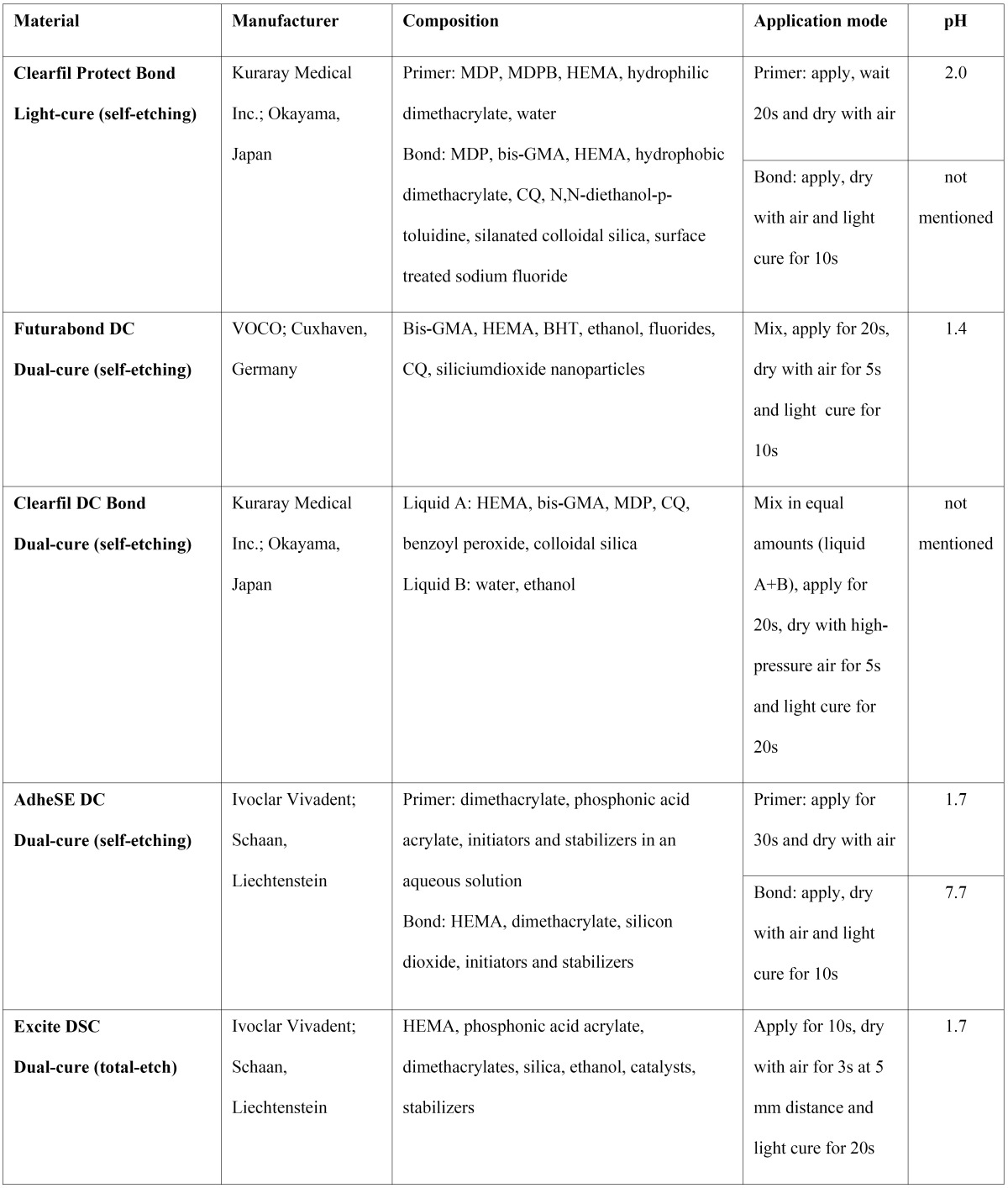
Adhesive systems tested. Composition, application mode and pH values.

The *E. faecalis* ATCC 29212 strain was taken from a 4ºC stock culture and streaked out twice on BHI (Scharlau Chemie S.A., Barcelona, Spain) agar plates for 24 hours at 37ºC.

-Biofilm formation test

The biofilm model used in this study was the MBEC–highthroughput (HTP) device (Innovotech, Edmonton, Alberta, Canada) (12), used in previous studies for E. faecalis (13,14). This batch-culture apparatus has a lid with 96 pegs that fits over a standard 96-well microtiter plate (15). Six pegs were coated with each AS tested ( Table 1), following the manufacturers instructions and 12 uncoated pegs served as the positive control (n=6) and the sterility control (n=6). The peg lid was then sterilized in ethylene oxide. Each assay was performed in duplicate for a total of twelve replicates per AS.

From a subculture of *E. faecalis*, a 1 McFarland standard *E. faecalis* suspension was prepared in BHI and then diluted 30-fold. The wells of a 96-well microtiter plate (Nunclon Delta Surface; Nunc, Roskilde, Denmark) were inoculated with 150 µL of the 1 in 30 dilution (approximately 1×107 CFU/mL), while 6 wells were inoculated with sterile BHI for the sterility control. The coated peg lid was fitted inside the wells, and the device was then placed on a rocking table (Swing Sw 8 10000-00015. OVAN, Badalona, Spain) at 5 rocks per minute, for 24 hours of incubation, at 37ºC and with 95% relative humidity. The cultures were checked for purity by Gram stain and colony morphology. Biofilms formed on the pegs were rinsed twice by placing the lid on two microtiter plates with 200 µL 0.9% saline solution in each well for 2 minutes to remove loosely adherent planktonic bacteria. The lid was then transferred to a microtiter recovery plate with 200 µL of BHI/well and sonicated on a water-table sonicator (Model 5510E–MT; Branson, Danbury, CT) for 10 minutes to disrupt the biofilm structure. The viability of the biofilms was determined by spot plating 10-µL aliquots of recovery biofilms onto BHI agar and incubating for 24 hours at 37ºC.

-Roughness test

Each material was applied on the flat surface of a microtiter plate. Roughness measurements were performed with a Mitutoyo 201 profilometer (Mitutoyo, Tokyo, Japan). Mean values (Ra, μm) were obtained from 13-15 measurements per material.

Statistical Analysis

Each comparison between two materials was performed using the Mann-Whitney test, and multiple comparisons by the Kruskal-Wallis test, both at a significance level of P < 0.05. The possible relationship between roughness and biofilm formation was established by lineal determination coefficient.

## Results

 Table 2 gives the mean values for the formation of *E. faecalis* biofilm and the roughness for each AS. The least amount of biofilm was found on Excite DSC, Clearfil Protect Bond and the polystyrene control, without statistically significant differences, followed in effectiveness by AdheSE DC and Clearfil DC Bond. The greatest amount of biofilm was obtained on Futurabond DC. In terms of roughness, the polystyrene control was the least rough, followed by Excite DSC and Clearfil DC Bond. Systems Clearfil Protect Bond and Futurabond DC gave the highest roughness values, with no statistically significant difference between the two. When excluding Clearfil Protect Bond because of its antimicrobial composition, a high linear correlation (R2 = 0.927) is observed between biofilm formation and roughness for the control and the rest of the AS.

**Table 2:**
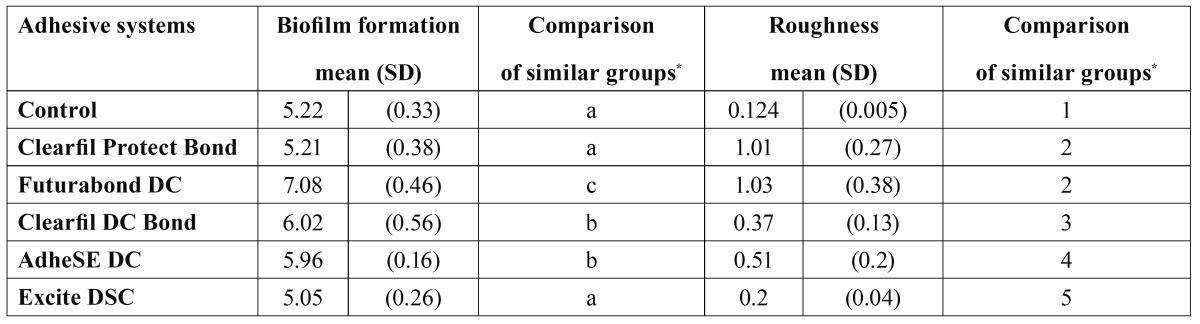
Comparison of E. faecalis biofilm formation on dentin adhesive systems and roughness. Mean (SD).

## Discussion

One promising approach to prevent microleakage in endodontic treatment may be the application of dentin adhesive materials, which can seal and protect root canal walls (16,17). Here, five commercially available AS were evaluated to test *E. faecalis* biofilm formation on their surface. Although dual-cure AS assure better polymerization in the deeper region of the root canal system, a light-cure adhesive (Clearfil Protect Bond) was used as a reference, given that it is an antibacterial dentin-bonding resin that effectively reduces the surface attachment of some bacteria strains such as S. mutans (18). *E. faecalis* was selected because it is commonly found in several situations such as in root canals of failing endodontically treated cases (19) as well as in chronic refractory periodontitis (20).

In order to study the biofilm formation on the surface of the five AS tested, the MBEC-HTP device was considered appropriate method because it allows for the simultaneous formation of 96 biofilms under similar conditions. This *in vitro* biofilm model is easy to use and permits aseptic manipulation of the samples (12).

The shear force created by the rocking table motion furthermore facilitates the formation of biofilms that are statistically equivalent (12,15). The biofilms were left to grow for 24 hours, considered an adequate incubation period for *E. faecalis* biofilm density (21). The results of this study indicate that *E. faecalis* biofilms formed upon all five AS tested, and for three the amounts were significantly greater than the control.

Biofilms are strongly influenced by some peculiar physical characteristics of the material, especially roughness (22), probably because surface irregularities protect bacteria against shear forces during their initial reversible adherence and provide a greater surface area for colonization. In our study, the peg-lid coated with the AS created significantly less smooth surfaces than the control. If Clearfil Protect Bond is excluded from the analysis, a high lineal correlation can be seen between roughness and biofilm formation. Excite DSC gave a roughness as low as the control, and the least amount of biofilm grew upon it; and Futurabond DC had the highest roughness and accumulated the greatest amount of biofilm. This relationship was not observed for Clearfil Protect Bond, which may be attributed to the MDPB molecule it contains, which would become immobilized after the adhesive polymerization (6,23) though still allowing a long-lasting antibacterial effect (24).

This finding confirms that factors besides the physical ones can influence the formation of biofilm, such as the chemical composition of AS (25) and its degree of conversion after curing. In fact, Futurabond DC, is an all-in-one AS with relatively low degrees of conversion (26,27). These systems contain high concentrations of hydrophilic resins and solvents and more water is trapped within the adhesive layer after curing, representing areas of increased permeability that would favor biofilm growth (26). The greater permeability would favor the formation of biofilm on its surface; and this, together with the high roughness demonstrated here, could explain the remarkably high values obtained for biofilm on the Futurabond DC surface.

AdheSE DC and Clearfil DC Bond gave congruent results in the tests, in the sense that their roughness is very similar and it lies in the intermediate range (0.51 and 0.37, respectively). Biofilm formation on these surfaces was also in the intermediate range and gave no statistical differences between the two systems.

An important goal in endodontic treatment is to eliminate the residual bacteria as well as impede the formation of biofilm in the root canal system. Different factors may influence the degree of success to this regard, such as pretreatment of the root dentin (28), the final irrigation treatment used (29), or the utilization of adhesive systems (30). Therefore, it would be desirable to formulate dual-cure adhesive systems that incorporate antimicrobial molecules such as MDPB, so that they might reduce biofilm formation and/or bacterial penetration in the filled root canal.

E. faecalis biofilms grew on the surface of all of the adhesives systems tested. The least amount of biofilm was obtained upon Clearfil Protect Bond which contains an antimicrobial molecule, and Excite DSC, which showed the least roughness. Futurabond DC, with high roughness, was the adhesive system that accumulated the most amount of biofilm. More research is needed to evaluate the potential with this approach.
